# Dairy products and calcium intake during pregnancy and dental caries in children

**DOI:** 10.1186/1475-2891-11-33

**Published:** 2012-05-17

**Authors:** Keiko Tanaka, Yoshihiro Miyake, Satoshi Sasaki, Yoshio Hirota

**Affiliations:** 1 Department of Preventive Medicine and Public Health, Faculty of Medicine, Fukuoka University, Fukuoka, 814-0180, Japan; 2 Department of Social and Preventive Epidemiology, School of Public Health, The University of Tokyo, Bunkyo-ku, Japan; 3 Department of Public Health, Osaka City University Graduate School of Medicine, Osaka, Japan

**Keywords:** Calcium, Dairy products, Dental caries, Prospective study

## Abstract

**Background:**

Maternal nutrition status during pregnancy may affect fetal tooth development, formation, and mineralization, and may affect dental caries susceptibility in children. We investigated the association between maternal intake of dairy products and calcium during pregnancy and the risk of childhood dental caries.

**Methods:**

Subjects were 315 Japanese mother-child pairs. Data on maternal intake during pregnancy were assessed through a diet history questionnaire. Outcome data was collected at 41–50 months of age. Children were classified as having dental caries if one or more primary teeth had decayed or been filled.

**Results:**

Higher maternal cheese intake during pregnancy was significantly inversely associated with the risk of dental caries in children, showing a clear inverse dose–response relationship; the adjusted odds ratio (OR) in comparison of the highest tertile with the lowest was 0.37 (95 % confidence interval [CI]: 0.17-0.76, *P* for trend = 0.01). The inverse associations between maternal intake of total dairy products, yogurt, and calcium during pregnancy and the risk of childhood dental caries were of borderline significance: the adjusted ORs for the highest tertile of total dairy products, yogurt, and calcium were 0.51 (95 % CI: 0.23-1.09, *P* for trend = 0.07), 0.51 (95 % CI: 0.23-1.10, *P* for trend = 0.07), and 0.50 (95 % CI: 0.23-1.07, *P* for trend = 0.08), respectively. There was no evident relationship between maternal milk intake and the risk of childhood dental caries.

**Conclusion:**

These data suggested that high intake of maternal cheese during pregnancy may reduce the risk of childhood dental caries.

## Background

Dental caries is the most common chronic disease of childhood worldwide. Though not life threatening, caries can cause pain and discomfort and reduce food intake, thereby affecting children’s quality of life [1]. Dental caries is a multi-factorial disease that is affected by physical and biological factors (morphology and composition of teeth, cariogenic bacteria, and fluoride exposure), lifestyle and behavioral factors (oral hygiene practices and dietary habits), and social status [2,3].

Primary tooth formation and mineralization starts during fetal development. Therefore the intrauterine environment, including maternal nutritional status, might play an important role in tooth development, formation, and mineralization [4,5]. Previous studies on the association between nutritional status and dental caries have mostly focused on malnutrition, and have shown that malnutrition affects tooth development and eruption and results in an increased rate of dental caries later in life [6-8]. Little is known, however, about the effects of maternal dietary intake during pregnancy on children’s dental health. In order to reduce the incidence of dental caries, greater knowledge not only of the deleterious effects of malnutrition but also of the beneficial effects of appropriate maternal dietary intake on dental health is needed.

The importance of calcium intake during pregnancy has been suggested with regard to caries prevention in children. To our knowledge, two epidemiological studies have addressed the association between maternal calcium supplementation during pregnancy and caries in children [9,10]^.^ A follow-up study of a randomized controlled trial in Argentina showed that maternal calcium supplementation during pregnancy was significantly inversely associated with dental caries in children at 12 years of age [9]. In another longitudinal study in Thailand, the incremental risk of caries between 9 and 12 months of age was lower among children whose mothers had received calcium supplements during pregnancy [10]. The purpose of this prospective study was to investigate the association between maternal intake of dairy products and calcium during pregnancy and the risk of dental caries in young Japanese children, using data from the Osaka Maternal and Child Health Study (OMCHS).

## Methods

### Study population

The OMCHS was a prospective cohort study to investigate risk and preventive factors for maternal and child health problems. Details of the baseline survey of the OMCHS, which was conducted during pregnancy, have been described elsewhere [11]. In brief, initially, only pregnant women who lived in Neyagawa City, one of the 43 municipalities in Osaka Prefecture, a metropolis in Japan, were recruited. Of the 3,639 eligible pregnant women in Neyagawa City, 627 (17.2 %) participated in the OMCHS between November 2001 and March 2003. Later, in order to increase the sample size, 375 pregnant women living in municipalities other than Neyagawa City were also recruited. Ultimately, a total of 1,002 pregnant women between the 5th and 39th week of pregnancy gave their fully informed consent in writing and completed the baseline survey. Of these 1,002 females, the numbers of participating individuals who took part in subsequent surveys were as follows:

2^nd^ survey 2–9 months postpartum 867

3^rd^ survey 16–24 months postpartum 763

4^th^ survey 29–39 months postpartum 586

5^th^ survey 41–49 months postpartum 494

Out of the 494 mother-child pairs who participated in all five surveys, 318 children received oral examinations when they were between 41 and 50 months of age. The current study was restricted to children whose mothers provided complete information on the variables under study, leaving data on 315 children available for analysis. The ethics committee of the Osaka City University School of Medicine approved the study.

### Outcome variable

Visual oral examinations between 41 and 50 months of age were performed by dental hygienists. The number of dental caries was recorded as the number of decayed or filled primary teeth (dft). The reasons for missing teeth were not identified in the OMCHS. Therefore, in the present study, children were classified as having dental caries if one or more primary teeth had decayed or been filled.

### Exposure variables and covariates

The baseline survey consisted of a set of two self-administered questionnaires. In addition, a self-administered questionnaire was used at the time of each follow-up survey, with participants mailing completed questionnaires to the data management center. Research technicians completed or corrected missing or illogical data by conducting telephone interviews with individual participants.

One of the self-administered questionnaires at baseline was a semi-quantitative, comprehensive diet history questionnaire (DHQ) that assessed dietary habits during the preceding month [12,13]. Estimates of daily intake of foods (150 items in total), energy, and selected nutrients were calculated using an ad hoc computer algorithm for the DHQ, based on the Standard Tables of Food Composition in Japan [14,15]. Total dairy product intake was defined as the sum of milk, yogurt and cheese intake. Information on dietary supplements was not used due to the lack of a reliable composition table for dietary supplements in Japan. Also, only a small number of participants (6.0 %) used calcium supplements at least once per week. According to a validation study of 92 females aged 31–69 years, Pearson’s correlation coefficient between the DHQ and 16-day weighted dietary records was 0.51 for calcium (unpublished data). Energy-adjusted intake by the residual method was used for the analyses [16].

A second questionnaire at baseline asked about maternal age, gestation, family income, maternal and paternal educational levels, and smoking habits.

The second survey consisted of a self-administered questionnaire that elicited information about the baby’s sex. The third survey consisted of a self-administered questionnaire that included questions about breastfeeding duration and the age in months at which solid foods were introduced. Breastfeeding duration was defined as the length of the period during which infants received breast milk, regardless of exclusivity. The fourth survey consisted of a questionnaire that elicited information about the age in months at first tooth eruption and about toothbrushing frequency in the child. In the fifth survey, each participant filled out a set of two self-administered questionnaires. One of the self-administered questionnaires was a brief diet history questionnaire that assessed the dietary habits of the child over the previous month. Mothers of children were asked to state how frequently their children consumed each of 51 selected food and non-alcoholic beverage items. Total dairy products intake by children was defined as the sum of milk, yogurt, and cheese intake. A second questionnaire included questions about toothbrushing frequency in the child, use of fluoride, pattern of professional dental care, and household smoking. The use of fluoride was defined as positive if mothers reported that their children used fluoride agents, such as toothpastes or gels. Household smoking was defined as positive if someone in the household reported smoking cigarettes.

### Statistical analyses

Intake of the dietary factors under study was categorized into tertiles based on its distribution in 315 subjects. Maternal age and gestation at the time of the baseline survey, family income, maternal and paternal educational levels, maternal smoking during pregnancy, the child’s gender, duration of breastfeeding, age when solid foods were introduced, age at first tooth eruption, toothbrushing frequency in the child as reported in the fourth and fifth surveys, use of fluoride, pattern of dental check-ups, household smoking as reported in the fifth survey, age at oral examination, and children’s total dairy products intake frequency were *a priori* selected as potential confounding factors.

Logistic regression analysis was performed to estimate crude odds ratios (ORs) and 95 % confidence intervals (CIs) for dental caries according to the tertile of intake of the dietary factors under investigation, with the lowest tertile as the reference. Multiple logistic regression analysis was employed to adjust for potential confounding factors. To test for linear trends across the tertiles of maternal intake of dairy products and calcium, we assigned a median value for the study population to each category and used these values as a continuous variable. Two-sided *P* values less than 0.05 were considered statistically significant. All statistical analyses were performed using the SAS software package, version 9.2 (SAS Institute, Cary, NC, USA).

## Results

There were no material differences between the 687 children whose mothers participated in the baseline survey of the OMCHS but who were excluded from the present study and the 315 study subjects with regard to distribution of gestation at baseline or maternal intake of total energy, milk, or cheese during pregnancy. Compared with the 687 children who were excluded from the present analysis, the 315 study subjects were more likely to have older mothers, report higher family income, have parents with relatively high educational levels, and have high intake levels of yogurt and calcium, while they were less likely to have been exposed to maternal smoking during pregnancy (Table 1).

**Table 1:** Characteristics and distribution of daily intake of dairy products and calcium at baseline in the study population (n = 315) as compared with the non-participating or excluded subjects (n = 687), OMCHS, Japan

**Variable**	**Study population, No (%) or mean (SD)**	**Excluded subjects, No (%) or mean (SD)**	***P*value**
**Baseline characteristics**			
Maternal age (years)			0.0003
< 29	91 (28.9)	289 (42.1)	
29-31	106 (33.7)	193 (28.1)	
≥ 32	118 (37.5)	205 (29.8)	
Gestational age at baseline (weeks)			0.23
< 15	111 (35.2)	246 (35.8)	
15-20	94 (29.8)	235 (34.2)	
≥ 21	110 (34.9)	206 (30.0)	
Family income (yen/year)			0.005
< 4,000,000	75 (23.8)	226 (32.9)	
4,000,000-5,999,999	129 (41.0)	274 (39.9)	
≥ 6,000,000	111 (35.2)	187 (27.2)	
Maternal education (years)			<0.0001
< 13	69 (21.9)	254 (37.0)	
13-14	136 (43.2)	277 (40.3)	
≥ 15	110 (34.9)	156 (22.7)	
Paternal education (years)			0.002
< 13	102 (32.4)	298 (43.4)	
13-14	55 (17.5)	117 (68.0)	
≥ 15	158 (50.2)	272 (39.6)	
Maternal smoking during pregnancy	43 (13.7)	141 (20.5)	0.009
**Daily food and nutrition intake**^**1**^			
Total energy (kJ)	7564.7 ± 1770.0	7689.8 ± 2054.8	0.32
Total dairy products (g)	180.8 ± 113.1	164.2 ± 124.8	0.04
Milk (g)	129.5 ± 101.4	119.2 ± 112.6	0.15
Yogurt (g)	45.5 ± 38.6	39.6 ± 46.8	0.04
Cheese (g)	5.9 ± 8.9	5.4 ± 7.9	0.40
Calcium (mg)	561.9 ± 168.6	529.5 ± 174.7	0.006

^1^Nutrient intake and food intake were adjusted for total energy intake using the residual method in a total of 1002 subjects.

Of the 315 children in the cohort, 74 (23.5 %) had developed dental caries by the time of the fifth survey, between 41 and 50 months of age. The mean number of decayed or filled primary teeth (dft) was 0.87. Approximately 14 % of the mothers indicated that they had smoked cigarettes during pregnancy (Table 2). Toothbrushing two or more times per day was reported at the fourth and fifth surveys in 36 % and 47 % of the subjects, respectively. Agents containing fluoride, such as toothpastes or gels, were used by approximately 83 % of the children, and approximately 40 % of the children received regular dental check-ups. Household smoking as reported during the fifth survey occurred in 37.1 % of our study subjects’ families. Maternal mean daily total energy consumption was 7564.7 kJ, and energy-adjusted intake of total dairy products and calcium were 179.4 g, and 556.1 mg, respectively (Table 3).

**Table 2:** Distribution of selected characteristics in 315 mother-child pairs, OMCHS, Japan

**Variable**	**Number (%)**
**Baseline characteristics**	
Maternal age (years)	
< 29	91 (28.9)
29-31	106 (33.7)
≥ 32	118 (37.5)
Gestational age at baseline (weeks)	
< 15	111 (35.2)
15-20	94 (29.8)
≥ 21	110 (34.9)
Family income (yen/year)	
< 4,000,000	75 (23.8)
4,000,000-5,999,999	129 (41.0)
≥ 6,000,000	111 (35.2)
Maternal education (years)	
< 13	69 (21.9)
13-14	136 (43.2)
≥ 15	110 (34.9)
Paternal education (years)	
< 13	102 (32.4)
13-14	55 (17.5)
≥ 15	158 (50.2)
Maternal smoking during pregnancy	43 (13.7)
**Characteristics at follow-up surveys**	
Child’s gender (male)	174 (55.2)
Breastfeeding duration (months)	
< 12	151 (47.9)
≥ 12	164 (52.1)
Age at introduction to solid foods (months)	
< 6	177 (56.2)
≥ 6	138 (43.8)
Age at first tooth eruption (months)	
< 7	157 (49.8)
≥ 7	158 (50.2)
Toothbrushing frequency in children at fourth survey (times/day)	
< 2	202 (64.1)
≥ 2	113 (35.9)
Toothbrushing frequency in children at fifth survey (times/day)	
< 2	168 (53.3)
≥ 2	147 (46.7)
Use of fluoride	
No	55 (17.5)
Yes	260 (82.5)
Regular dental check-ups	127 (40.3)
Household smoking at fifth survey	117 (37.1)
Age at oral examination (months)	
< 43	190 (60.3)
≥ 43	125 (39.7)
Children’s dairy products intake at fifth survey (times/week)	12.3 (8.6)

**Table 3:** Distribution of daily intake of dairy products and calcium in 315 pregnant women at baseline

**Variable**	**Mean (SD)**
Total energy (kJ)	7564.7 ± 1770.0
Total dairy products (g)	179.4 ± 113.0
Milk (g)	128.6 ± 101.2
Yogurt (g)	45.0 ± 38.6
Cheese (g)	5.8 ± 8.9
Calcium (mg)	556.1 ± 168.3

Nutrient intake and food intake were adjusted for total energy intake using the residual method.

Pearson’s correlation coefficients between maternal intake of milk, yogurt, and cheese and total calcium intake were 0.74 (95 % CI: 0.68-0.78), 0.43 (95 % CI: 0.33-0.52), and 0.38 (95 % CI: 0.28-0.47), respectively.

Compared with the lowest tertile of maternal cheese intake during pregnancy, the highest tertile was significantly inversely associated with the risk of dental caries in children, showing a clear inverse dose–response relationship (the adjusted OR for the highest tertile = 0.37, 95 % CI: 0.17-0.76, *P* for trend = 0.01) (Table 4). The inverse associations between maternal intake of total dairy products and yogurt during pregnancy and the risk of dental caries in children were of borderline significance: the adjusted ORs for the highest tertiles compared with the lowest tertiles of total dairy products and yogurt intake were 0.51 (95 % CI: 0.23-1.09, *P* for trend = 0.08) and 0.51 (95 % CI: 0.23-1.10, *P* for trend = 0.07), respectively. No evident relationship was observed between maternal milk intake during pregnancy and the risk of dental caries in children. Maternal calcium intake during pregnancy tended to be inversely associated with the risk of dental caries in children: the adjusted OR for the highest tertile versus the lowest was 0.50 (95 % CI: 0.23-1.07, *P* for trend = 0.08).

**Table 4:** Odds ratios and 95 % confidence intervals for dental caries according to tertile of maternal intake of dairy products and calcium during pregnancy in 315 children, OMCHS, Japan

**Variable**	**Tertile of intake**			
	**1**	**2**	**3**	***P*for trend**
**Total dairy products**				
Intake (g/day)^1^	82.0	171.0	264.6	
Range	−72.8 − 122.2	122.3 − 204.7	204.8 − 582.7	
Risk	31/105 (29.5 %)	21/105 (20.0 %)	22/105 (21.0 %)	
Crude OR (95 % CI)	1.00	0.60 (0.31, 1.12)	0.63 (0.33, 1.18)	0.15
Adjusted OR (95 % CI)^2^	1.00	0.58 (0.28, 1.18)	0.51 (0.23, 1.09)	0.08
**Milk**				
Intake (g/day)^1^	31.6	123.4	182.7	
Range	−82.2 − 80.1	80.2 − 153.8	153.9 − 468.0	
Risk	28/105 (26.7 %)	23/105 (21.9 %)	23/105 (21.9 %)	
Crude OR (95 % CI)	1.00	0.77 (0.41, 1.45)	0.77 (0.41, 1.45)	0.39
Adjusted OR (95 % CI)^2^	1.00	0.74 (0.36, 1.49)	0.64 (0.29, 1.37)	0.23
**Yogurt**				
Intake (g/day)^1^	7.9	36.6	90.1	
Range	−14.3 − 17.8	17.9 − 62.3	62.4 − 167.5	
Risk	29/105 (27.6 %)	25/105 (23.8 %)	20/105 (19.1 %)	
Crude OR (95 % CI)	1.00	0.82 (0.44, 1.52)	0.62 (0.32, 1.17)	0.15
Adjusted OR (95 % CI)^2^	1.00	0.97 (0.47, 1.99)	0.51 (0.23, 1.10)	0.07
**Cheese**				
Intake (g/day)^1^	0.5	3.5	10.1	
Range	−7.7 − 2.1	2.2 − 4.8	4.9 − 90.4	
Risk	37/105 (35.2 %)	22/105 (21.0 %)	15/105 (14.3 %)	
Crude OR (95 % CI)	1.00	0.49 (0.26, 0.90)	0.31 (0.15, 0.59)	0.001
Adjusted OR (95 % CI)^2^	1.00	0.56 (0.27, 1.14)	0.37 (0.17, 0.76)	0.01
**Calcium**				
Intake (mg/day)^1^	408.1	535.4	690.3	
Range	99.5 − 476.6	476.7 − 607.4	607.5 − 1221.6	
Risk	31/105(29.5 %)	22/105 (21.0 %)	21/105 (20.0 %)	
Crude OR (95 % CI)	1.00	0.63 (0.33, 1.18)	0.60 (0.31, 1.12)	0.12
Adjusted OR (95 % CI)^2^	1.00	0.65 (0.31, 1.31)	0.50 (0.23, 1.07)	0.08

^1^ Values for intake are medians adjusted for energy intake by the residual method for each tertile.

^2^ Adjusted for maternal age at baseline survey, gestation at baseline, family income, maternal and paternal educational levels, maternal smoking during pregnancy, child’s gender, breastfeeding duration, age at introduction to solid foods, age at first tooth eruption, toothbrushing frequency in children at fourth and fifth surveys, use of fluoride, pattern of dental check-ups, household smoking at fifth survey, age at oral examination, and children’s dairy products intake at fifth survey.

To examine whether the association of maternal intake of total dairy products, yogurt, and cheese during pregnancy with the risk of dental caries in children could be attributed to calcium intake, we conducted an additional analysis in which we adjusted for maternal calcium intake during pregnancy as a continuous variable. In this analysis, the inverse association of maternal cheese intake during pregnancy with the risk of dental caries in children was essentially unaltered after further adjustment for maternal intake of calcium during pregnancy: the additionally adjusted ORs from the lowest intake tertile to highest were 1.00 (reference), 0.57 (95 % CI: 0.27-1.16), and 0.40 (0.18-0.86), respectively (*P* for trend = 0.03). Conversely, the marginally significant inverse associations between intake of total dairy products and yogurt in the highest tertile and the risk of dental caries in children were attenuated after further adjustment for maternal calcium intake: the additionally adjusted ORs were 0.58 (95 % CI: 0.19-1.70, *P* for trend = 0.33) and 0.58 (95 % CI: 0.25-1.32, *P* for trend = 0.18), respectively.

## Discussion

In this prospective study in Japan, we found that higher maternal intake of cheese during pregnancy was associated with a decreased risk of dental caries in children. Higher maternal intake of total dairy products, yogurt, and calcium during pregnancy tended to be associated with a reduced risk of dental caries in children, although there was no evident association between maternal milk intake and the risk of childhood dental caries. To our knowledge, this is the first study to assess the possible inverse association between maternal dietary calcium intake during pregnancy and dental caries in children. Our results are in partial agreement with previous findings showing an inverse relationship between maternal calcium intake by supplement during pregnancy and dental caries in children [9,10].

The potential protective effects of maternal intake of total dairy products and yogurt during pregnancy on caries in children were attenuated when we additionally controlled for maternal calcium intake during pregnancy in this study. Therefore, the beneficial association between maternal intake of total dairy products and yogurt and dental caries in children may be ascribed to some extent to calcium intake or to unmeasured constituents in relation to calcium. Primary tooth formation and mineralization usually begins at the 13th week of gestation. Higher maternal calcium intake during pregnancy might influence tooth mineralization, causing tooth enamel to be more acid-resistant [10]. Additionally, teeth are known to accumulate lead during development [17]. Both animal and human studies have shown that teeth with high lead levels are generally more susceptible to dental caries [17]. Ettinger *et al.*[18] have demonstrated that calcium supplementation during pregnancy was associated with reduced blood lead levels. Maternal lead level might therefore be another important factor affecting the mineralization of teeth.

In the current study, the risk reduction associated with maternal intake of cheese during pregnancy did not appear to be confounded by calcium intake. We have no immediate explanation for the potential mechanisms underlying the observed inverse association between cheese intake and dental caries. Thus components of cheese other than calcium might be responsible for the protective effects of maternal cheese intake against dental caries in children. Alternatively, high maternal intake of cheese may simply reflect a healthier diet and/or lifestyle in general. The observed beneficial effect of maternal cheese intake on dental caries in children might therefore be spurious. In addition, high maternal intake of cheese during pregnancy may reflect high intake of cheese by children after the introduction of solid foods. Intake levels of foods and nutrients are strongly correlated between mothers and children [19,20], and some cross-sectional studies have shown a significant inverse association between cheese intake and the prevalence of dental caries [21,22]. It is possible that both prenatal and postnatal cheese intake are protective against childhood dental caries. In the present study, however, the Pearson’s correlation coefficients between maternal intake of energy-adjusted milk, yogurt, and cheese during pregnancy and children’s intake frequency of milk, yogurt, and cheese at the fifth survey were 0.22 (95 % CI: 0.11-0.32), 0.16 (95 % CI: 0.05-0.26), and 0.08 (95 % CI: -0.03-0.19), respectively. Thus maternal dairy products intake during pregnancy is likely to have little relation to the children’s frequency of dairy products intake in the present study. Our previous cross-sectional study among Japanese children aged 3 years found an inverse association between intake of yogurt, but not cheese or milk, and the prevalence of dental caries in children [23]. This is at variance with our present findings. Our observed results may be merely a consequence of multiple testing and/or chance.

Our study has methodological strengths. The prospective design probably reduced the likelihood of recall bias, and we were able to control for relevant confounding factors. However, we cannot completely exclude the possibility that unknown or residual confounding factors have biased our data.

There are limitations to be considered as well. First, our DHQ could only approximate consumption. The most likely effect of this would an underestimation of values in our results. Our DHQ was designed to assess recent dietary intake, specifically, for one month prior to completing the questionnaire. It is possible that the pregnant women in this study had departed from their typical diets for reasons such as nausea gravidarum. However, Cucó *et al.*[24] have shown that dietary patterns remain fairly constant throughout pregnancy. Therefore, food intake information obtained at one point during pregnancy is likely to reliably represent food intake throughout pregnancy.

Second, selection bias could have affected our results. Of the 1,002 participants at baseline, only 315 children (31.4 %) were included in this analysis. Moreover, at baseline, the participation rate for those living in Neyagawa City was only 17.2 % of the total number of pregnant mothers. In other areas, the participation rate could not be calculated because the exact numbers of eligible subjects among the populations from which participants were recruited were not available. Thus, the children in this study are probably not truly representative of the general population. In fact, educational levels were higher in the parents in our study than in the general population [25]. In addition, only 23.5 % of our children had dental caries at 41–50 months of age. According to the National Survey of Dental Disease conducted in 2005 [26], the prevalence of dental caries in their sample of 4-year-old Japanese children was 44.2 %. The population we surveyed might have maintained a greater awareness concerning oral health than the general population has, and this could have influenced the results. With regard to dietary intake, calcium intake in this study population is similar to that in the general population. According to the National Health and Nutrition Survey in Japan, the average daily per capita intake of calcium was 531.1 mg [27], whereas the mean daily intake of our study subjects was 556.1 mg.

Third, we might have had insufficient power to detect possible associations given the small number of study subjects. It is also possible that the small degree of variation in intake of dairy products among Japanese people helped to obscure the associations between maternal intake of dairy products during pregnancy and the risk of dental caries in children. According to the National Health and Nutrition Survey in Japan, the average daily per capita intake of milk and dairy products was 123.9 g [27]. In the US between 1999 and 2004, in contrast, the average daily per capita consumption of dairy products by women was 240 g [28]. The difference in consumption of dairy products between Japanese and Western populations should be taken into account when interpreting our results. A clear protective association of dairy products and calcium with dental caries may exist in populations with high intake of dairy products and calcium. The results obtained in the present study, therefore, are not likely to be generalized worldwide.

Fourth, oral examinations were performed by dental hygienists. The dental hygienists were given detailed criteria for performing the examinations, but they received no specific training in standardizing their examinations. Additionally, since radiography was not employed, approximal lesions may have been underdiagnosed, leading to non-differential misclassification of caries, and thus a bias toward the null.

Our results suggested that high intake of maternal cheese during pregnancy may reduce the risk of dental caries in children. Higher intake of total dairy products, yogurt, and calcium during pregnancy tended to be associated with a lower risk of dental caries in children. Further studies are needed to replicate our findings and to clarify the mechanisms underlying the possible inverse associations between maternal intake of dairy products and calcium and the risk of dental caries in children.

## Abbreviations

CI, confidence interval; DHQ, diet history questionnaire; OMCHS, Osaka Maternal and Child Health Study; OR, odds ratio.

## Competing interests

The authors declare that they have no competing interests.

## Authors’ contributions

KT contributed to data acquisition, data management, statistical analysis, data interpretation, and manuscript writing. YM contributed to the study design and the data acquisition. SS contributed to the study design. YH supervised the study design and execution. All authors read and approved the final manuscript.
